# Are 3D-printed Models of Tibial Plateau Fractures a Useful Addition to Understanding Fractures for Junior Surgeons?

**DOI:** 10.1097/CORR.0000000000002137

**Published:** 2022-02-28

**Authors:** Jellina M. Huitema, Nynke van der Gaast, Lars Brouwers, Ruurd L. Jaarsma, Job N. Doornberg, Michael J. R. Edwards, Erik Hermans, , and the Traumaplatform 3D Consortium

**Affiliations:** 1 Department of Trauma Surgery, Radboud University Medical Centre and Radboud University, Nijmegen, the Netherlands; 2 Department of Trauma Surgery, Elizabeth–Tweesteden Ziekenhuis, Tilburg, the Netherlands; 3 Department of Orthopaedic and Trauma Surgery, Flinders Medical Centre and Flinders University, Adelaide, Australia; 4 Department of Orthopaedic Surgery, University Medical Centre Groningen and Groningen University, Groningen, the Netherlands

## Abstract

**Background:**

Tibial plateau fractures are often complex, and they can be challenging to treat. Classifying fractures is often part of the treatment process, but intra- and interobserver reliability of fracture classification systems often is inadequate to the task, and classifications that lack reliability can mislead providers and result in harm to patients. Three-dimensionally (3D)-printed models might help in this regard, but whether that is the case for the classification of tibial plateau fractures, and whether the utility of such models might vary by the experience of the individual classifying the fractures, is unknown.

**Questions/purposes:**

(1) Does the overall interobserver agreement improve when fractures are classified with 3D-printed models compared with conventional radiology? (2) Does interobserver agreement vary among attending and consultant trauma surgeons, senior surgical residents, and junior surgical residents? (3) Do surgeons’ and surgical residents’ confidence and accuracy improve when tibial plateau fractures are classified with an additional 3D model compared with conventional radiology?

**Methods:**

Between 2012 and 2020, 113 patients with tibial plateau fractures were treated at a Level 1 trauma center. Forty-four patients were excluded based on the presence of bone diseases (such as osteoporosis) and the absence of a CT scan. To increase the chance to detect an improvement or deterioration and to prevent observers from losing focus during the classification, we decided to include 40 patients with tibial plateau fractures. Nine trauma surgeons, eight senior surgical residents, and eight junior surgical residents—none of whom underwent any study-specific pretraining—classified these fractures according to three often-used classification systems (Schatzker, OA/OTA, and the Luo three-column concept), with and without 3D-printed models, and they indicated their overall confidence on a 10-point Likert scale, with 0 meaning not confident at all and 10 absolutely certainty. To set the gold standard, a panel of three experienced trauma surgeons who had special expertise in knee surgery and 10 years to 25 years of experience in practice also classified the fractures until consensus was reached. The Fleiss kappa was used to determine interobserver agreement for fracture classification. Differences in confidence in assessing fractures with and without the 3D-printed model were compared using a paired t-test. Accuracy was calculated by comparing the participants’ observations with the gold standard.

**Results:**

The overall interobserver agreement improved minimally for fracture classification according to two of three classification systems (Schatzker: κ_conv_ = 0.514 versus κ_3Dprint_ = 0.539; p = 0.005; AO/OTA:κ_conv_ = 0.359 versus κ_3Dprint_ = 0.372; p = 0.03). However, none of the classification systems, even when used by our most experienced group of trauma surgeons, achieved more than moderate interobserver agreement, meaning that a large proportion of fractures were misclassified by at least one observer. Overall, there was no improvement in self-assessed confidence in classifying fractures or accuracy with 3D-printed models; confidence was high (about 7 points on a 10-point scale) as rated by all observers, despite moderate or worse accuracy and interobserver agreement

**Conclusion:**

Although 3D-printed models minimally improved the overall interobserver agreement for two of three classification systems, none of the classification systems achieved more than moderate interobserver agreement. This suggests that even with 3D-printed models, many fractures would be misclassified, which could result in misleading communication, inaccurate prognostic assessments, unclear research, and incorrect treatment choices. Therefore, we cannot recommend the use of 3D-printed models in practice and research for classification of tibial plateau fractures.

**Level of Evidence:**

Level III, diagnostic study.

## Introduction

Tibial plateau fractures are common [4, 6, 8, 20], and they can be devastating [9, 17]. Classifying fractures often is part of the treatment process, but many classifications used for this task are unreliable [16]. Classifications that lack reliability can result in erroneous prognostic estimates, misleading research, unclear or inaccurate communication among providers, and treatment choices that may harm patients. Currently, radiographs, two-dimensional (2D) CT, and three-dimensional (3D) reconstructions are used to classify fractures and plan surgery [2, 3, 5, 19, 21]. 3D-printed models are seeing wider use because of increased availability and decreased material costs; costs can be as low as USD 10 for a model [22].

The value of 3D-printed models in classifying and assessing fractures is controversial. Studies on intraarticular distal radius fractures and distal humerus fractures show that 3D-printed models do not improve interobserver agreement [1, 11]. A similar study on acetabular fractures demonstrated that 3D-printed models improve interobserver agreement in classification compared with conventional radiographs, but not compared with CT [13]. However, in another study on acetabular fractures [2], interns, residents, and junior surgeons had a higher interobserver agreement for classification with a 3D-printed model than senior surgeons, with the greatest benefit from 3D-printed models in surgical interns. This suggests that there might be some clinical value of 3D models for training and education of residents in orthopaedic trauma surgery. However, to our best knowledge, there are no studies on the additional value of 3D-printed models for the interobserver agreement of fracture classifications of tibial plateau fractures. Furthermore, to our knowledge, there are no studies evaluating the difference in interobserver agreement between surgeons and surgical residents regarding tibial plateau fractures.

While comparing additional 3D-printed models with conventional radiology, we aimed to answer three main study questions: (1) Does the overall interobserver agreement improve when fractures are classified with 3D-printed models compared with conventional radiology? (2) Does interobserver agreement vary among attending and consultant trauma surgeons, senior surgical residents, and junior surgical residents? (3) Do surgeons’ and surgical residents’ confidence and accuracy improve when tibial plateau fractures are classified with an additional 3D model compared with conventional radiology?

## Patients and Methods

### Study Design

Based on the hospital’s registration system for the diagnosis of tibial plateau fracture, 113 adult patients (≥ 18 years) with a tibial plateau fracture who were treated between June 2012 and June 2020 were identified for this observational study. Forty-four patients without a good-quality preoperative CT scan and those with a bone disease (such as osteoporosis or osteogenesis imperfecta) were excluded. Previous studies on the additional value of 3D-printed models were based on approximately 20 fractures and could not demonstrate a clinically relevant improvement [2, 11]. To ensure that observers could stay focused during the classification process and that a possible clinically relevant improvement could be achieved, we decided to include 40 patients with a tibial plateau fracture. Two nonobserver authors (JMH, NvdG) screened fractures before inclusion to ensure that the chosen fractures capture the main characteristics of the used classification systems. According to the Schatzker classification, we included two Type I plateau fractures, 13 Type II, two Type III, five Type IV, two Type V, and 16 Type VI.

Furthermore, we ensured that the different fracture types, according to the Schatzker classification, were equally divided into two groups by using a random number generator: a 3D-printed model group and a conventional radiology group (Table 1).

**Table 1.** Overview of the number of subtypes of the Schatzker classification in each group

Schatzker type	3D-printed model group	Conventional radiology group
I	1	1
II	6	7
III	1	1
IV	3	2
V	1	1
VI	8	8

### Image Viewer

Digital Imaging and Communications in Medicine (DICOM) images of all fractures were retrieved using KPACS (version 1.6.0, IMAGE Information Systems Europe GmbH). DICOM files were anonymized using MATLAB® (version R2020a, the MathWorks Inc). The DICOM files of all radiographs, 2D CT images, and 3D CT images were presented using a Philips DICOM viewer (R3.0-SP03, Philips Medical Systems Nederland BV), Windows Photo Viewer, and Windows Media Player (Windows 7, Microsoft Corp).

### 3D-printed Models

In the 3D-printed group, CT DICOM images with an axial cut thickness of 0.5 mm were obtained from KPACS and imported into 3D Slicer (version 4.10.1) [7]. We used a threshold between 180 and 400 Hounsfield units to identify the tibial plateau and its fracture fragments. To remove unwanted floating voxels, we used the island removal algorithm and the scissor and eraser tool. To create a smooth 3D printed model without small interruptions, we used the paint tool and a closing algorithm. All 3D models were exported as surface tessellation language files into Ultimaker Cura software (version 4.6.1, Ultimaker BV). The surface tessellation language files were exported to a 3D printer (Ultimaker S5, Ultimaker BV) and printed with polylactic alcohol as the construction material and polyvinyl alcohol as a water-soluble support structure. The following preprocessing parameters were used: layer height, 0.2 mm; infill density, 15%; print speed, 45 mm/s; and extruder temperature 215° C to 225° C for polylactic acid and polyvinyl alcohol.

### Participants and Fracture Classification

Nine trauma surgeons, eight senior surgical residents, and eight junior surgical residents from seven different Level I, II, and III trauma centers in the Netherlands were asked to participate as observers. The trauma surgeons had a median (range) work experience of 19 years (6 to 32). In the Netherlands, trauma surgery is most commonly performed by general surgeons who specialize in trauma, and therefore surgical residents participated in this study. The senior surgical residents were in their final year of surgical training and all specialized in trauma surgery. The participating junior surgical residents were in their first year of general surgical training. The observers did not receive any study specific pretraining before the assessment; however, they did have access to a visual representation of each classification system.

**Fig. 1:**
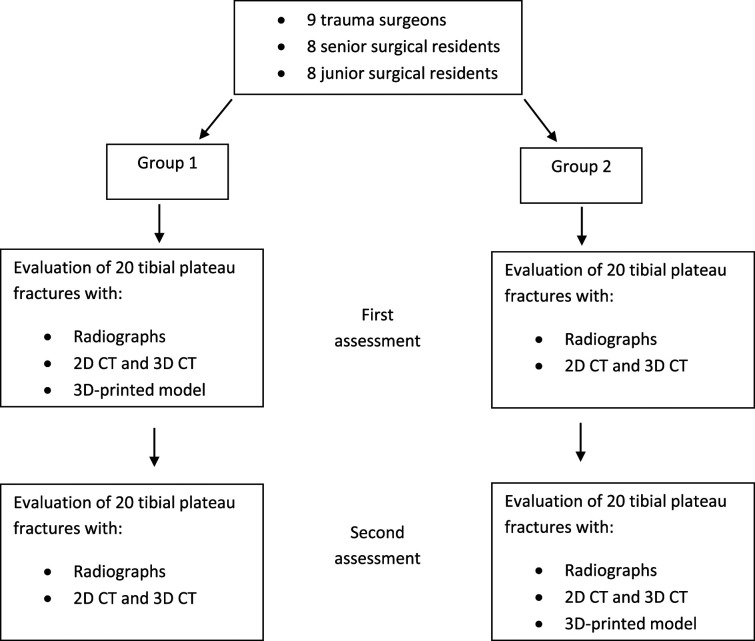
This flowchart shows the study’s design.

All observers were anonymized and subsequently divided into two groups to achieve an equal distribution of trauma surgeons, senior surgical residents, and junior surgical residents. At the first assessment, the first group received radiographs, 2D CT images, and 3D CT images with an additional 3D-printed model. The second group received only conventional radiography (radiographs and 2D and 3D CT images). During the second assessment, the first group received conventional radiography and the second group received conventional radiography with an additional 3D-printed model (Fig. 1). The assessments occurred with at least 1 month between them to prevent learning bias.

From the five classification systems for tibial plateau fractures that have been tested for inter- and intraobserver reliability, three classification systems that represent a different level of simplicity were chosen for this study [16]: the Schatzker classification [18], the AO/OTA classification [15], and the Luo three-column concept [14]. Each observer was asked to classify the fractures according to these three systems. After viewing each fracture, participants indicated their overall confidence regarding their fracture assessment on a 10-point Likert scale, with 0 meaning not confident at all and 10 meaning completely confident.

To define a gold standard, a panel of three experienced trauma surgeons who had special expertise in knee surgery and 10 years to 25 years of experience in practice (EH, MJRE, and SvdG, a member of the Traumaplatform 3D consortium) classified all fractures according to the Schatzker classification, the AO/OTA classification, and the Luo three-column concept until consensus was reached. During this assessment, the surgeons had full access to preoperative radiographs, 2D and 3D CT images, 3D-printed models, operative reports, and postoperative radiographs of all fractures.

Data were collected using a questionnaire created in Castor EDC (v2020.2.11, Castor EDC). During fracture assessment, one of the authors (JMH, NvdG) was present to provide radiographs, CT images, and 3D-printed models of fractures and complete the questionnaire in Castor EDC.

### Ethical Approval

We obtained approval from our institutional review board to perform this study.

### Statistical Analysis

We used the Fleiss κ to determine the interobserver agreement. The κ value is a chance-corrected quantitative measure representing the degree to which observers agree with each other. To interpret κ values, Landis and Koch [10] proposed the following division: a κ between 0.01 and 0.20 reflects slight agreement, between 0.21 and 0.40 reflects fair agreement, between 0.41 and 0.60 reflects moderate agreement, between 0.61 and 0.80 reflects substantial agreement, and greater than 0.81 reflects almost-perfect agreement. Differences between the κ values were calculated. The values were considered significant when the two-tailed p value of the difference was less than 0.05.

Furthermore, accuracy was determined by comparing fracture classifications by the observers with the gold standard set by the expert panel, expressed as a percentage. Differences in confidence in fracture classification, with and without the 3D model, were compared using a paired t-test. A two-tailed p value less than 0.05 was considered significant. All statistical analyses were performed using IBM SPSS Statistics, version 25, and Microsoft Office Excel 2016. There were no missing values.

## Results

### Overall Interobserver Agreement

Overall, 3D-printed models of tibial plateau fractures did not lead to categorical changes in any of the three classification systems. The Schatzker and AO/OTA classification showed a small improvement in interobserver agreement with the addition of 3D print models (Schatzker: κ_conv_ = 0.514 versus κ_3Dprint_ = 0.539; p = 0.005; AO/OTA:κ_conv_ = 0.359 versus κ_3Dprint_ = 0.372; p = 0.03). No differences in agreement were found in the Luo three-column concept (κ_conv_ = 0.514 versus κ_3Dprint_ = 0.532; p = 0.08) (Table 2).

**Table 2.** Overview of the overall interobserver agreement for conventional radiology versus 3D printed models

Classification	κ for conventional radiology	Agreement^a^	κ for the 3D model	Agreement^a^	Difference of κ (95% CI)	p value
Schatzker	0.514	Moderate	0.539	Moderate	-0.025 (- 0.042 to -0.008)	0.005
AO/OTA	0.359	Fair	0.372	Fair	-0.013 (-0.025 to -0.001)	0.03
Luo three-column	0.514	Moderate	0.532	Moderate	-0.018 (-0.038 to 0.002)	0.08

a Agreement proposed by Landis and Koch [10].

### Interobserver Agreement Among Trauma Surgeons, Senior Surgical Residents, and Junior Surgical Residents

For junior surgical residents and senior surgical residents, 3D-printed models of tibial plateau fractures did not result in categorical changes in any of the three classification systems. Trauma surgeons demonstrated a categorical deterioration in the AO/OTA classification (κ_conv_ = 0.426 versus κ_3Dprint_ = 0.338; p < 0.001). For trauma surgeons, no differences in agreement were found in the Schatzker classification and the Luo three-column concept (Schatzker: κ_conv_ = 0.576 versus κ_3Dprint_ = 0.556; p = 0.44; Luo: κ_conv_ = 0.506 versus κ_3Dprint_ = 0.555; p = 0.09) (Table 3). For senior surgical residents, 3D-printed models of tibial plateau fractures led to a small improvement in interobserver agreement in the Schatzker classification (κ_conv_ = 0.494 versus κ_3Dprint_ = 0.551; p = 0.04). There were no differences in agreement in the AO/OTA classification and the Luo three-column concept (AO/OTA: κ_conv_ = 0.372 versus κ_3Dprint_ = 0.372; p = 0.99; Luo: κ_conv_ = 0.574 versus κ_3Dprint_ = 0.554; p = 0.56) (Table 4). For junior surgical residents, 3D-printed models led to an improvement in interobserver agreement in all three classification systems (Schatzker: κ_conv_ = 0.443 versus κ_3Dprint_ = 0.566; p < 0.001; AO/OTA: κ_conv_ = 0.282 versus κ_3Dprint_ = 0.324; p = 0.03; Luo: κ_conv_ = 0.433 versus κ_3Dprint_ = 0.548; p < 0.001) (Table 5).

**Table 3.** An overview of the interobserver agreement between trauma surgeons on conventional radiology versus 3D printed models

Classification	κ for conventional radiology	Agreement^a^	κ for the 3D model	Agreement^a^	Difference in κ (95% CI)	p value
Schatzker	0.576	Moderate	0.556	Moderate	0.020 (-0.032 to 0.073)	0.44
AO/OTA	0.426	Moderate	0.338	Fair	0.088 (0.053 to 0.123)	< 0.001
Luo three-column	0.506	Moderate	0.555	Moderate	-0.048 (-0.105 to 0.009)	0.09

a Agreement proposed by Landis and Koch [10].

**Table 4.** Overview of the interobserver agreement between senior surgical residents for conventional radiology versus 3D printed models

Classification	κ for conventional radiology	Agreement^a^	κ for the 3D model	Agreement^a^	Difference in κ (95% CI)	p value
Schatzker	0.494	Moderate	0.551	Moderate	-0.058 (-0.113 to -0.002)	0.04
AO/OTA	0.372	Fair	0.372	Fair	0.000 (-0.040 to 0.040)	> 0.99
Luo three-column	0.574	Moderate	0.554	Moderate	0.020 (-0.047 to 0.087)	0.56

a Agreement proposed by Landis and Koch [10].

**Table 5.** Overview of the interobserver agreement between junior surgical residents on conventional radiology versus 3D printed models

Classification	κ for conventional radiology	Agreement^a^	κ for the 3D model	Agreement^a^	Difference in κ (95% CI)	p value
Schatzker	0.443	Moderate	0.566	Moderate	-0.123 (-0.178 to -0.069)	< 0.001
AO/OTA	0.282	Fair	0.324	Fair	-0.042 (-0.081 to -0.003)	0.03
Luo three-column	0.433	Moderate	0.548	Moderate	-0.115 (-0.183 to -0.047)	< 0.001

a Agreement proposed by Landis and Koch [10].

### Accuracy and Confidence

Neither the overall accuracy nor the accuracy for all subgroups (trauma surgeons and senior and junior surgical residents) improved in any classification system when 3D printed models were used (Table 6). Furthermore, there was no overall improvement or improvement in the different subgroups in self-assessed confidence for classifying fractures with additional 3D-printed models. The overall self-assessed confidence was a 7 of 10.

**Table 6.** Overview of the accuracy of fracture classification using conventional radiology versus 3D printed models

Classification	Overall for conventional radiololgy	Overall for the 3D model	Conventional radiology ofor surgeons	3D model for surgeons	Conventional radiology for senior surgical residents	3D model for senior surgical residents	Conventional radiology for senior surgical residents	3D model for junior surgical residents
Mean accuracy for Schatzker classification	68%	66%	71%	68%	68%	64%	66%	66%
Mean accuracy for the AO/OTA classification	52%	51%	57%	51%	50%	56%	48%	47%
Mean accuracy for the Luo classification	59%	72%	60%	72%	63%	74%	54%	71%

Accuracy = expressed as a percentage of correctly classified fractures compared with the gold standard (expert panel).

## Discussion

Classifications are often unreliable and can therefore result in treatment choices that may harm patients. By using new techniques, such as 3D-printed models, orthopaedic surgeons try to improve the classification process. However, studies on humerus, distal radius, and acetabular fractures show no improvement in interobserver agreement. The additional value of 3D-printed models in the classification of tibial plateau fractures remained dubious and was therefore evaluated in this study. In line with previous studies, we found only small improvements of the interobserver agreement, and no classification system achieved more than moderate interobserver agreement [1, 11, 13]. This suggests that even with the addition of 3D-printed models, many fractures would be misclassified, leading to misleading communication, inaccurate prognostic assessments, and incorrect treatment choices.

### Limitations

This study has several limitations. First, we found minimal improvement in interobserver agreement; however, no interobserver agreement better than moderate was achieved. This improvement could be explained by learning bias, especially for junior surgical residents, as they are the least experienced observers. We tried to diminish this learning bias by providing a minimum time period of 1 month between the first and second assessment and providing a crossover between the two observer groups. A surprising result is the deterioration in the interobserver agreement for trauma surgeons in the AO/OTA classification. This suggests that, even with an additional 3D-printed model, trauma surgeons would misclassify more fractures than without this additional information. This highlights the poor additional value of classification systems in the preoperative planning of tibial plateau fractures. In contrast with the low interobserver agreement, we found a high self-assessed confidence in all subgroups. Given that we only asked the overall confidence instead of the confidence per classification system, we cannot analyze this further by classification system.

### Discussion of Key Findings

Overall, additional 3D-printed models led to a small improvement of interobserver agreement for two of three classification systems. However, we did not find any categorical changes resulting from use of these models, and none of the classifications achieved more than moderate agreement. In a study on the additional value of 3D-printed models in distal radius fractures, no improvement in interobserver agreement was found, and in line with our findings, all interobserver agreements were slight to moderate, except for one substantial interobserver agreement [11]. The study of Lim et al. [13] on acetabular fractures even found no interobserver agreement more than fair. These results, together with our study, suggest that most of the fractures have been misclassified, even with the help of 3D-printed models, and therefore the reliability of these classification systems in clinical practice or research is insufficient.

When using 3D-printed models, we found that junior surgical residents had the greatest improvement in interobserver agreement, senior surgical residents showed intermediate improvement, and trauma surgeons showed no improvement. None of the observer groups achieved more than moderate interobserver agreement. In accordance with our findings, Brouwers et al. [2] and Lim et al. [13] showed that junior surgical residents had the highest improvement in interobserver agreement in classifying acetabular fractures and no interobserver agreement more than moderate was achieved. Although their results may indicate otherwise, residents in the study of Lim et al. [13] indicated a positive experience with 3D-printed models. In line with this, a systematic review on the use of 3D-printed models in surgical teaching identified that 3D-printed models were found to be superior to all forms of 2D anatomy teaching [12]. Questionnaires on 3D-printed models in general and integrating 3D-printed models in surgical education were overwhelmingly positive. 3D-printed models may not lead to a clinically relevant improvement in interobserver agreement of classification systems, nevertheless they could be valuable in surgical education.

In contrast to previous studies [2, 13], our study showed no improvement in observer confidence and accuracy in assessing tibial plateau fractures with 3D-printed models. The high self-assessed confidence in all groups forms a big contrast with the low interobserver agreement and low accuracy. This suggests that observers’ confidence is misplaced and that even experienced trauma surgeons seem to be unaware of their capability of classifying tibial plateau fractures. The misplaced confidence compared with the low interobserver agreement could lead to inaccurate prognostic assessments, incorrect treatment choices, and misleading communication for patients with tibial plateau fractures.

### Conclusion

3D-printed models do not lead to a clinically relevant improvement in interobserver agreement for the AO/OTA classification, Schatzker classification, or the Luo three-column concept, and therefore, they offer no additional value in the classification of tibial plateau fractures compared with conventional radiographs and CT scans. Furthermore, in this study, we did not record any instances of interobserver agreement more than moderate, which suggests that a high number of tibial plateau fractures would be misclassified when using these three classification systems. This could result in incorrect treatment of patients with tibial plateau fractures, more posttraumatic complications, and a longer recovery. Therefore, we cannot recommend the use of models in the classification of tibial plateau fractures.

## Group Authors

Members of the Traumaplatform 3D Consortium include: Kamil van der Velde, Tiemen van Boxtel, Yannick ’t Mannetje, David Reetz, Kyrill Rykov, Hendrike Bolkenstein, Rutger Stijns, Simon Yauw, Martijn de Kruijf, Steven Strang, Hugo Fokkenrood, Mandala Leliveld, Matthijs ter Horst, Rian Wijkmans, Pieter Hoogland, Joost Peters, Jan Biert, Bas van Wageningen, Jan Paul Frölke, Erik van der Krol, Edwin. Dierselhuis, Micha Holla, Edo Hekma, Karel Kolkman, and Sebastiaan van de Groes.
